# Intelligence

**Published:** 1828-04

**Authors:** 


### INTELLIGENCE.

### MONTHLY REPORT OF PREVALENT DISEASES.

DURING the early part of the present month, the weather was so extremely mild as to leave us few of the diseases of winter. Latterly, however, the easterly winds and diminished temperature have produced abundance of more acute cases and inflammations about the chest; rheumatism and catarrhs have been very rife. The former has been particularly severe and obstinate, but has yielded sooner to large doses of calomel and opium than to any other remedies.

Among individual cases, the only one worth mentioning is that of a young woman who had swallowed about half an ounce of oxalic acid a fortnight previously. Free vomiting was excited soon after the poison had been taken, but pain at the pit of the stomach continued more or less constant. At the time of her application to us, there was pretty general tenderness of the abdomen, with a frequent, small, but not hard pulse; and the bowels were much confined. She has done well under the employment of small bleedings, local and general, and frequent doses of castor oil.

### *Bye-Laws of the College of Physicians relative to the Licentiates.*

DE PERMISSIS.

1. STATUIMUS et ordinamus ut nemo in Permissorum numerum eligendus proponatur, qui non annum ætatis suæ vicesimum sextum se clausisse Præsidentem et Censores, in Comitiis Minoribus,* certiores fecerit, et qui non gradum Doctoris in Medicinâ susceperit, et, antequam illum gradum susceperit, per duos annos integros in aliquâ Academiâ animo medicinæ studendi

* Comitia Minora dicuntur, quæ habentur à Præsidente et Censoribus, cum aliàs, quoties ipsis visum fuerit, tum primo die Veneris, singulis mensibus, quo die Examinationes institui solent in ædibus Collegii. Comitia Majora a sociis universis celebrantur.

commoratus fuerit, et commorationis istius literas testimoniales in illâ Academiâ usitatas Comitiis Minoribus exhibuerit.

2. Nemo in Permissorum numerum admittatur qui medicamentum quodvis arcanum (*nostrum* vulgò dictum) in morbis curandis usurpare solitus fuerit; nisi ante examinationem primam id medicamentum, ac ejusdem adhibendi modum, Præsidenti aut Propræsidenti et Censoribus plane exposuerit.

3. Antequàm quispiam in Permissorum numerum admittatur, si fortè Chirurgorum aut Pharmacopolarum sodalitio olim donatus fuerit, sodalitii istius privilegiis omnibus renunciet, nec non emancipationis suæ literas firmâ auctoritate comprobatas Registrario proferat.

4. Nemo in Permissorum numerum admittatur qui non prius examinatus et approbatus fuerit, in tribus Comitiis minoribus, secundum hanc formam:—

Unusquisque eorum qui in numerum Permissorum admitti petat, examinetur:—

In Primis Comitiis, in parte Medicinæ Physiologicâ.

In Secundis, in parte Pathologicâ.

In Tertiis, in parte Therapeuticâ.

Præterea in singulis Examinationibus, locum è Celso, vel è Sydenhami operibus, Anglicè reddat. Singulæ Examinationes prædictæ Latinè fiant.

5. Qui ad hanc formam in Comitiis minoribus examinatus et in singulis examinationibus approbatus fuerit, in Comitiis majoribus proximè insequentibus proponatur in Permissorum numerum admittendus, et si major pars Sociorum præsentium consenserit, quamprimum admittatur.

6. Si vero quispiam in unâ quâvis Examinationum prædictarum, minus peritus, nec ad Medicinæ facultatem in urbe Londino et intra septem milliaria in circuitu ejusdem exercendam idoneus existimatus fuerit, non nisi præterito integro anno ad Examinationem iterum admittatur.

7. Antequam quispiam in numerum Permissorum admittatur, det fidem infra-scriptam Præsidenti aut Propræsidenti, coram Sociis in majoribus Comitiis:—

"Dabis fidem te observaturum statuta Collegii, aut mulctas tibi contra facienti irrogandas promptè persoluturum, omniaque in Medicinâ faciendâ pro viribus facturum in honorem Collegii et Reipublicæ utilitatem."

8. Admittendus flexis genubus,* (*eodem modo quo Socii,*) manus invicem applicatas humiliter tradat in manus Præsidentis vel Propræsidentis, qui dicat:—

"Ego *A. B.* Præsidens, vel Propræsidens, hujus Collegii admitto te ad Medicinæ facultatem in urbe Londino et per septem milliaria in circuitu ejusdem exercendam quamdiu te bene gesseris, precorque tibi omnia fausta."

9. Si quis vero in urbe Londino, aut per septem milliaria in circuitu ejusdem, Medicinæ facultatem exercuerit, qui ad hoc per Præsidentis et Collegii literas sigillo nostro communi sigillatas non sit admissus; auctoritate Præsidentis et Censorum, si modo ipsis aut eorum majori parti ita visum fuerit, admoneatur per schedulam in manus traditam, vel in domo ejus relictam, ut medicinam exercere desinat, donec examinatus et approbatus fuerit, literis ad hoc datis sigillo Collegii munitis.

Qui monitum hoc neglexerit, legibus regni obnoxius erit.

* Si quis religionis causâ genua flectere nolit, hoc prætermitti potest.

DE ELECTIONE EXTRAORDINARIA IN PERMISSOS ET IN SOCIOS.

1. Quandoquidem fieri potest, ut viri sint quidam egregii et in studiis medicis satis versati, qui non duos annos integros ante susceptum gradum Doctoris Medicinæ in aliquâ Academiâ commorati fuerint, ideoque per statutum nostrum de Permissis in numerum Permissorum admitti vetentur, statuimus et ordinamus ut non obstante statuto de Permissis, liceat Præsidenti pro suo arbitrio quotannis unum Medicinæ Doctorem, utpote ob morum integritatem et artis medicæ peritiam, in numerum Permissorum approbandum Censoribus proponere. Et si, Examinationibus tribus ordinariis pro Permissis institutis debitè peractis, approbatus fuerit, liceat Præsidenti illum in numerum Permissorum eligendum Collegio proponere, et si major pars Sociorum præsentium consenserit, in numerum Permissorum quamprimum admittatur.

2. Liceat etiam Præsidenti quotannis unum pro suo arbitrio è Permissis, qui decennium compleverit à tempore admissionis, utpote morum integritate, doctrinâ, et artis medicæ peritiâ insignem, in Socium proponere; et si major pars Sociorum præsentium consenserit, in Societatem nostram quamprimum admittatur.

3. Liceat porrò cuilibet Sociorum in Comitiis majoribus ordinariis, postridie divi Michaelis habendis, aliquem qui annos septem integros in numero Permissorum fuerit, annumque ætatis suæ tricesimum sextum clauserit, examinandum proponere, et si, Examinationibus ordinariis pro Candidatis institutis in tribus Comitiis majoribus ordinariis debitè peractis, approbatus fuerit, et major pars Sociorum præsentium consenserit, in Societatem nostram quamprimum commodè fieri potest, admittatur.

DE CONVERSATIONE MORALI ET STATUTIS PŒNALIBUS.

1. Nullus sive Socius, sive Candidatus, sive Permissus fuerit, Socium aut Candidatum aut Permissum ignorantiæ in arte suâ vel maleficii nomine, nisi coram judicibus legitimis accuset, aut coram quibusvis afficiat contumeliis. Si quem contrà fecisse Præsidenti et Censoribus aut eorum majori parti innotuerit, primâ vice solvat quatuor libras, secundâ vice duplicetur mulcta; quòd si tertio quis similiter deliquerit, et modo prædicto convictus fuerit, si quidem Socius aut Candidatus fuerit, expellatur è Societate nostrâ, vel è Candidatorum ordine; sin idem sit è Permissorum numero, solvat decem libras. Quam quidem decem librarum mulctam quotiescunque idem Permissus ejusdem delicti modo prædicto denuò convictus fuerit, ipsi irrogandam statuimus.

2. Nullus Socius, Candidatus, vel Permissus salutatione officiosâ, vel animi benevoli obtentu, opem medicam ultrò offerat, nedum subministret ægro cuilibet, quem Medici cujusvis, sive Socii, sive Candidati, sive Permissi, curæ commissum esse cognoverit, et ad quem non accersitus fuerit.

3. Si quis autem malitiæ hujusmodi convictus fuerit, præter ignominiæ notam quam isti (quantum in nobis est) inuri volumus, quadraginta solidos mulctetur à Presidente et Censoribus.

4. Si quis paciscatur cum Pharmacopolis de aliquâ pretii parte ex medicamentis præscribendis percipiendâ, si sit Socius aut Candidatus, et hujusce delicti à Præsidente et majore parte Sociorum in Comitiis majoribus sive ordinariis sive extraordinariis præsentium convictus fuerit, è Societate nostrâ, vel è Candidatorum ordine, expellatur.

5. Sin Permissus delicti hujusce à Præsidente et Censoribus, aut eorum majore parte, convictus fuerit, decem libras quotiescunque id admiserit, mulctetur.

6. Medicus quisque, sive Socius, sive Candidatus, sive Permissus fuerit, singulis suis schedulis, in quibus ægri curatio præscribitur, diem præscriptionis, ægri nomen, et sui denique nominis literas initiales adscribat; nisi causa intersit à Præsidente et Censoribus approbanda.

7. Si plures Medici curationis gratiâ convenerint, consultandùm est summâ modestiâ, et non nisi semotis arbitris à re alienis. Nec quisquam præscribat, imò ne innuat quidem, quid agendum sit, corum ægro, aut adstantibus, priusquam junctis consiliis inter ipsos Medicos curandi methodus fuerit constituta, Sin autem Medici in diversas iverint sententias, ita ut in eandem medendi methodum consentire nequeant, summâ tamen prudentiâ et moderatione se gerant; eorumque dissentionem ita, ut tàm ægro quàm amicis ejus quàm minimum molestiæ pariat, ordinarius medicus ægro aut adstantibus significet.

8. Qui leges has consultandi non observaverit, et à Præsidente et Censoribus aut eorum majore parte convictus fuerit, quinque libras mulctetur.

9. Nullus denique Medicus, sive Socius, sive Candidatus, sive Permissus, consilium ineat de rebus Medico propriis, in civitate Londino et intra septem milliaria in circuitu ejusdem nisi cum aliquo è Sociorum vel Candidatorum vel Permissorum numero, sub pœnâ quinque librarum quotiescunque hujusce delicti à Præsidente et Censoribus, aut eorum majore parte convictus fuerit.

10. Omnes mulctæ quæ per statuta nostra irrogatæ fuerint illico solvantur.

### DE PERMISSIS EXTRA URBEM.

1. Nemo ad Medicinam extra urbem Londinum et septem milliaria in circuitu ejusdem exercendam admittatur, qui non annum ætatis suæ vicesimum sextum clauserit, et non in aliquâ Academiâ, aut in aliquo Nosocomio Londinensi per annum integrum, aut in Nosocomio Provinciali per biennium, animo Medicinæ studendi commoratus fuerit, et præterea Prælectiones Anatomicas et Medicas audierit; de quibus omnibus Præsidentem, vel Propræsidentem, fecerit certiorem.

2. Antequàm quispiam in numerum Permissorum extra urbem admittatur, si fortè Chirurgorum aut Pharmacopolarum sodalitio olim donatus fuerit, sodalitii istius privilegiis omnibus renunciet.

3. Nemo in numerum Permissorum extra urbem admittatur qui non priùs a Præsidente et tribus Electis examinatus et approbatus fuerit, quemadmodum legibus regni cautum est.

4. Examinatio Latinè fiat.

*Imprimatur hic libellus, cui titulus* "Quæ ad Permissos pertinent," &c.

*Datum ex Ædibus Collegii in Comitiis Majoribus, Februarii Mensis 20mo*, 1828.

HENRICUS HALFORD, *Præses.*

THOMAS TURNER, JACOBUS TATTERSALL, FRANCISCUS HAWKINS, JOHANNES CARR BADELEY, } *Censores.*

---

*Copy of the last Report of the National Vaccine Establishment to the Secretary of State for the Home Department; presented pursuant to an Address of the House of Commons, bearing date the* 13*th February*, 1828.

To the RIGHT HONOURABLE ROBERT PEEL, Secretary of State for the Home Department.

SIR,—We have the honour to inform you, that the result of the last year's experience is highly favorable to vaccination, and that we hear most satisfactorily, not only of its protective influence, but of its wider diffusion.

1

It is true that cases are reported to us very often of the occurrence of small-pox after vaccination; but we have reason to believe that the number of those who fall into this safe, though sometimes severe, disease after vaccination, is not greater than that of those who formerly *died* by inoculation, whilst that practice prevailed.

With regard to the diffusion of this protection, whether we judge by the extent of the demand which has been made upon the Board for authentic lymph in the course of the last year, or collect from accounts received of the practice of vaccination in new countries, we are satisfied that the prejudices against it are less pertinacious than they were: and, where it is not resorted to with that alacrity and thankfulness which such a blessing might justly demand, the failure is rather to be attributed to a propensity in human nature to disregard danger at a distance, and to wait till the evil be at the door, before measures are taken to prevent it, than to a distrust of its saving influence.

In proof of its wider diffusion, we learn that it is now practised, not only throughout the Morea and the countries inhabited by the Greeks, but that it has been admitted into Constantinople, and into the palace of the Sultan, in spite of the prejudices which the religion of the Mahomedans opposes to any measure intended to interfere with the destinies of life. So that the advantages which this country derived from the East in the last century, by the acquisition of inoculation from thence, it has now abundantly requited by imparting to the same region the safer practice of vaccination, by which the small pox, equal to their own plague in the severity of its visitations, has been already disarmed of its terrors, and in the course of years may, possibly, be extinguished altogether. We have, &c.

HENRY HALFORD, *President of Roy. Coll. Physicians.*
THOMAS TURNER, } *Censors of the Royal College of*
JAMES TATTERSALL, } *Physicians.*
HENRY PASTON COOPER,
*President of the Royal College of Surgeons.*
ANTHONY CARLISLE,
*Vice-President of the Royal College of Surgeons.*
CLEMENT HUE, M.D. *Registrar.*

*National Vaccine Establishment, Percy-street;*
*13th February,* 1828.

---

*General Dispensary, Aldersgate-street.*—A vacancy in the appointment of Surgeon to this Institution has occurred, by the resignation of Mr. LLOYD. The candidates for the situation are Mr. W. COULSON, of the Medical School, Aldersgate-street; and Mr. C. CASWALL, house surgeon of St. Bartholomew's Hospital. The election is fixed for the 16th instant.

### METEOROLOGICAL JOURNAL,

*From February 20th, to March 20th, 1828.*

By Messrs. Harris and Co. Mathematical Instrument Makers, 50, High Holborn.

| February | Rain gauge. | Moon. | Thermom. 9 A.M. | Thermom. MAX. | Thermom. MIN. | Barometer. 9 A.M. | Barometer. 10 P.M. | De Luc's Hygrom. 9 A.M. | De Luc's Hygrom. 10 P.M. | Winds. 9 A.M. | Winds. 10 P.M. | Atmospheric Variations. 9 *a.m.* | Atmospheric Variations. 2 *p.m.* | Atmospheric Variations. 10 *p.m.* |
|---|---|---|---|---|---|---|---|---|---|---|---|---|---|---|
| 20 | | | 43 | 49 | 40 | 29.09 | 29.06 | 99 | 98 | SE | S | Cloudy | Fine | Rain |
| 21 | | | 42 | 45 | 39 | 28.94 | 28.93 | 99 | 98 | SE | ESE | — | — | Cloudy |
| 22 | | ☽ | 43 | 47 | 41 | 28.90 | 29.01 | 98 | 98 | SE | ESE | — | Rain | — |
| 23 | | | 41 | 42 | 39 | 29 20 | 29.42 | 98 | 98 | NE | N | Rain | Fair | — |
| 24 | | | 42 | 50 | 39 | 29.68 | 29.71 | 97 | 96 | WSW | WSW | Fair | Fine | Fair |
| 25 | | | 44 | 52 | 48 | 29.74 | 29.73 | 96 | 98 | WSW | WSW | — | Rain | — |
| 26 | | | 50 | 57 | 50 | 29.74 | 29.77 | 98 | 98 | WSW | WSW | — | Fair | — |
| 27 | | | 52 | 53 | 49 | 29.99 | 30.09 | 94 | 98 | SW | S | Cloudy | Cloudy | — |
| 28 | | | 50 | 55 | 40 | 30.20 | 30.21 | 97 | 96 | NE | NE | — | Fair | Cloudy |
| 29 | | | 42 | 47 | 41 | 30.12 | 29.93 | 98 | 98 | NW | ENE | Fair | — | — |
| Mar. | | | | | | | | | | | | | | |
| 1 | | ○ | 46 | 48 | 45 | 30.03 | 29.94 | 94 | 97 | N | N | — | — | Fair |
| 2 | | | 45 | 50 | 38 | 29.94 | 29.97 | 96 | 98 | ENE | NE | — | — | — |
| 3 | | | 42 | 48 | 42 | 29.88 | 29.76 | 95 | 92 | WNW | N | Cloudy | — | Cloudy |
| 4 | | | 43 | 50 | 40 | 29.84 | 29.63 | 92 | 93 | NW | N | Fair | — | Cloudy |
| 5 | | | 42 | 45 | 30 | 29.63 | 29.65 | 90 | 84 | NNE | N | Cloudy | Sleet | Fine |
| 6 | | | 35 | 40 | 31 | 29.65 | 29.92 | 76 | 90 | N | N | Fair | Fair | Cloudy |
| 7 | | | 33 | 38 | 31 | 30.11 | 30.03 | 80 | 98 | NW | W | — | — | Rain |
| 8 | | | 46 | 55 | 47 | 30.00 | 30.12 | 98 | 96 | NW | W | — | — | Cloudy |
| 9 | | ☾ | 48 | 57 | 45 | 30.13 | 30.10 | 94 | 90 | W | W | — | Fine | Fine |
| 10 | | | 53 | 58 | 44 | 30.10 | 30.11 | 90 | 92 | WNW | NW | — | — | — |
| 11 | | | 52 | 55 | 46 | 30.11 | 30.12 | 92 | 92 | W | WSW | — | — | Fair |
| 12 | | | 48 | 55 | 43 | 29.95 | 29.95 | 94 | 98 | WSW | WNW | Cloudy | — | Cloudy |
| 13 | | | 52 | 60 | 52 | 30.01 | 30.04 | 93 | 96 | W | WNW | Fine | — | — |
| 14 | | | 54 | 59 | 46 | 30.14 | 30.15 | 88 | 86 | W | WNW | — | — | — |
| 15 | | ● | 50 | 62 | 49 | 30.26 | 30.25 | 86 | 80 | N | NW | Cloudy | — | — |
| 16 | | | 55 | 62 | 52 | 30.22 | 30.16 | 92 | 99 | W | WNW | — | Fair | Sleet |
| 17 | | | 57 | 64 | 48 | 30.04 | 30.04 | 92 | 94 | W | NW | — | — | Fair |
| 18 | .15 | | 51 | 56 | 48 | 30.06 | 29.55 | 94 | 96 | NW | WNW | — | — | Sleet |
| 19 | | | 49 | 52 | 42 | 29.35 | 29.47 | 98 | 94 | W | WNW | — | Cloudy | Fine |

